# Persistent HIV-1 Viremia on Antiretroviral Therapy: Measurement and Mechanisms

**DOI:** 10.3389/fmicb.2019.02383

**Published:** 2019-10-15

**Authors:** Jana L. Jacobs, Elias K. Halvas, Melissa A. Tosiano, John W. Mellors

**Affiliations:** Division of Infectious Diseases, Department of Medicine, University of Pittsburgh School of Medicine, Pittsburgh, PA, United States

**Keywords:** HIV-1 persistence, HIV-1 cure, low-level viremia, antiretroviral therapy, plasma viremia, plasma HIV-1 RNA

## Abstract

HIV-1 viremia persists at low-levels despite clinically effective antiretroviral therapy (ART). Here we review new methods to quantify and characterize persistent viremia at the single genome level, and discuss the mechanisms of persistence including clonal expansion of infected cells and tissue origins of viremia. A deeper understanding of how viremia persists on ART is critically important to the design of therapies to eliminate viremia and achieve a functional cure for HIV-1.

## Introduction

Over the last 30 years there have been great strides made in the diagnosis and treatment of HIV-1 infection. The broad implementation of antiretroviral therapy (ART) has saved the lives of many millions of persons with HIV (Palella et al., 1998), but has also uncovered the persistence of HIV-1 on ART, both as a latent reservoir and as an expression of low-level viremia (Chun et al., 1995, 1997a,b; Finzi et al., 1997; Wong et al., 1997; Dornadula et al., 1999). Studies of patients on ART revealed two sources of plasma viremia: (1) short-lived, productively infected CD4+ T-cells that produce bursts of virus and then die; and (2) long-lived cells capable of producing viremia that is below the limit of detection of commercial assays (Ho et al., 1995; Wei et al., 1995; Perelson et al., 1996, 1997; Dornadula et al., 1999). Longitudinal studies of persistent plasma HIV-1 viremia below the limit of detection of commercial assays in persons on long-term ART have provided a biphasic model of viremia decay, including an initial decay phase with a half-life of 39 weeks and a more slowly decaying phase with a half-life of 11 years (Maldarelli et al., 2007; Palmer et al., 2008; Riddler et al., 2016). These studies showed a positive association between persistent plasma viremia and pre-ART plasma HIV-1 RNA. Riddler et al. (2016) reported that persistent plasma viremia was associated with higher CD8 T-cell counts and a lower CD4/CD8 ratio on ART, both markers of incomplete immune recovery. The source(s) and mechanisms of persistent HIV-1 plasma viremia are still largely uncharacterized despite improved detection methods. Because persistent viremia represents a major barrier to HIV-1 cure, its characterization and clearance remain a high priority. Here we review some recent advances in measuring and identifying the origins of persistent viremia.

## Persistent Hiv-1 Viremia on Art

### Measurement and Recent Improvements

The development of more sensitive, reverse-transcriptase initiated quantitative PCR (RT qPCR) assays revealed that more than half of individuals on ART with plasma HIV-1 RNA suppressed below the limit of detection of commercial assays (20–40 copies/ml) still have detectable HIV-1 RNA in plasma, averaging around 1–3 copies/mL (Dornadula et al., 1999; Palmer et al., 2003, 2008; Maldarelli et al., 2007; Zheng et al., 2013; Riddler et al., 2016). Recent innovations have improved the measurement of this low-level plasma HIV-1 RNA. The first-generation two-step qRT-PCR assay with single copy sensitivity targeted HIV-1 *gag* (gSCA) and required 6–7 mL of plasma (Palmer et al., 2003). A subsequent single copy qRT-PCR assay targeted a highly conserved region of integrase in HIV-1 pol (iSCA v1.0) and enhanced nucleic acid recovery from a smaller volume of plasma (Cillo et al., 2014). Despite highly successful implementation in many clinical studies, iSCA v1.0 required ultracentrifugation and only assayed about half of the total extracted nucleic acid for HIV-1 RNA. In the most recent iteration of a single-copy assay (iSCA v2.0), ultracentrifugation is replaced with tabletop centrifugation and a greater proportion (∼80%) of the total extracted nucleic acid is tested for HIV-1 RNA. Importantly, when equal volumes of the same donor plasma were tested using versions of iSCA, 55% of the samples that had no HIV-1 RNA detected by iSCA v1.0 had HIV-1 RNA detected by iSCA v2.0 (Tosiano et al., 2019a).

Automated, next-generation commercial platforms can reproducibly measure HIV-1 RNA in plasma above the limit of quantification (20–200 copies/mL depending on the platform) (Wiesmann et al., 2018). Although individual measurements using commercial platforms do not provide the sensitivity of manual single copy assays, automated platforms have potential as a screening tool. For example, results reported by either Roche or Abbott automated platforms as <20 or <40 copies/mL respectively (also known as “detected but not quantifiable”) are almost always detected and quantified by manual single copy assay (Margot et al., 2018; Tosiano et al., 2019b), whereas automated platform results indicating “no target detected” are associated with a significantly lower frequency of HIV-1 RNA detection by manual single copy assays. In addition, Bakkour et al. (2019) have reported that reasonable estimates of HIV-1 RNA copies/mL below the limit of quantification can be obtained using automated platforms to test multiple replicates of plasma to generate a combination of detected, non-detected, and detected but not quantifiable results. Each sample can be assigned a value for HIV-1 RNA by applying a mathematical algorithm based upon the qualitative readouts. Comparisons are in progress of HIV-1 RNA levels obtained by manual single copy assays versus multiple replicates on automated platforms. An automated platform with single copy sensitivity would have distinct advantages over more labor intensive and lower throughput, manual single copy assays.

### Association of Persistent Viremia With Cell-Associated HIV-1 DNA

The half-life of persistent plasma viremia on stable ART, calculated using decay dynamics modeling, is more than 11 years (Riddler et al., 2016). Interestingly, decay of HIV-1 proviral DNA-containing cells on ART was recently reported to have a similar half-life of 13 years (Gandhi et al., 2017). Although it is enticing to suggest that the similar half-lives of total cell-associated HIV-1 DNA and plasma HIV-1 RNA on ART represent a direct association between infected cells and persistent plasma viremia, it is important to note that proviral DNA-containing cells rarely contain full-length, intact proviruses. In fact, less than 1–10% of proviruses that persist on ART are capable of producing infectious virus (Fourati et al., 2012; Ho et al., 2013; Bruner et al., 2019). Despite this data, many have reported direct associations of varying degrees between qPCR measures of the proviral reservoir (total cell-associated HIV-1 DNA) and persistent plasma viremia, suggesting that they are related (Chun et al., 2011; Mexas et al., 2012; Hong et al., 2018). The recent development of an assay capable of quantifying intact proviral DNA (Intact Proviral DNA Assay, IPDA) will help address questions regarding the degree of correlation between total and intact cell-associated DNA and plasma viremia (Bruner et al., 2019). Intact proviral DNA correlated modestly with *ex vivo* measurements of inducible, infectious virus outgrowth. However, such quantitative viral outgrowth assays (qVOAs) have not correlated with levels of persistent plasma viremia in individuals on ART (Siliciano et al., 2003; Eriksson et al., 2013). qVOAs have also been shown to underestimate the size of the reservoir by missing the fraction of intact provirus that is non-inducible *ex vivo*; this fraction could contribute to plasma viremia *in vivo* (Ho et al., 2013; Bruner et al., 2019). As such, assays that quantify intact proviruses may show stronger correlations with plasma viremia than total HIV-1 DNA. Studies are currently in progress to assess this possibility.

### Association of Persistent Viremia With Cell-Associated HIV-1 RNA

Measurements of various forms of cell-associated bulk HIV-1 RNA have been used to estimate proviral transcriptional activity, both at steady-state and in response to latency reversal agents (Pasternak et al., 2008; Strain and Richman, 2013; Kiselinova et al., 2014; Kearney et al., 2015; Procopio et al., 2015; Hong et al., 2016; Li et al., 2016; Yukl et al., 2018). Whether cell-associated HIV-1 RNA correlates with production of plasma virus is debated. Typically, PCR-based approaches have targeted a single small region of bulk HIV-1 RNA. Although important information about HIV-1 pathogenesis has been garnered from these assays, their utility for assessing latency reversal or changes in viremia have been questioned (Eriksson et al., 2013; Archin et al., 2014; Elliott et al., 2014). In a recent study, cell-associated unspliced HIV-1 RNA strongly correlated with plasma viremia in untreated individuals, but not in individuals on ART (Hong et al., 2018). These conflicting results could be attributed to the accumulation of defective provirus after ART initiation (Bruner et al., 2016) leading to production of defective transcripts that do not result in virion production (Imamichi et al., 2016; Pollack et al., 2017; Wiegand et al., 2017). Interestingly, an analysis of various forms of cell-associated HIV-1 RNA transcripts that are expressed following treatment with latency reversal agents showed very different expression profiles depending upon the conditions and agent used, most of which did not lead to production of full-length polyadenylated transcripts (Yukl et al., 2018; Moron-Lopez et al., 2019). Given the uncertain value of measuring cell-associated HIV-1 RNA in all infected cells, assays that measure HIV-1 RNA transcribed only from intact proviruses (and therefore more likely to produce plasma virus) are likely to be more useful for assessing interventions aimed at perturbing and/or eliminating the HIV-1 reservoir (Wiegand et al., 2017; Yucha et al., 2017).

## Sources of Persistent Hiv-1 Viremia on Art

### Cell and Tissue Sources

Understanding the source of persistent viremia is critically important for the design of interventions to eliminate it. While the source of persistent viremia is likely multi-faceted and variable across individuals, ongoing studies are seeking common sources of viremia that could serve as more specific therapeutic targets. In virologically suppressed individuals, most HIV-1 proviral DNA is found in resting CD4+ T-cells (Chun et al., 1997a; Finzi et al., 1997; Wong et al., 1997). Though not considered a major contribution to the reservoir, other cells types such as monocytes, macrophages, and hematopoietic stem cells have been reported to harbor proviral DNA (Sonza et al., 2001; Zhu et al., 2002; Zaikos et al., 2018; Mitchell et al., 2019). The propensity for white blood cells to circulate throughout the body and penetrate various tissues and lymphoid organs provides an opportunity for infected cells to access ordinarily anatomically protected and/or immune-privileged sites. As such, HIV-1 RNA and/or DNA have been detected widely across tissues in virologically suppressed individuals, including in lymph nodes (Perreau et al., 2013), cerebrospinal fluid (Dahl et al., 2014; Spudich et al., 2019), adipose tissue (Couturier et al., 2015; Damouche et al., 2015), gut-associated lymphoid tissue (GALT) (Lampinen et al., 2000; Anton et al., 2003; Belmonte et al., 2007); and most recently, in the urethra (Ganor et al., 2019).

By contrast, the cellular and/or tissue reservoir(s) that contribute to persistent plasma viremia have not been clearly identified. It is possible that multiple sources contribute to variable degrees, and that sources vary between individuals. Historically, comparison of sequences of persistent plasma virus and total cell-associated proviral DNA in circulating CD4+ T-cells have rarely identified matches. This finding is likely because limited sequencing methods may not detect rare intact provirus that are producing virus. Indeed, in a study in which intensive sampling and single genome sequencing were performed, proviral sequences were detected that did match persistent plasma virus (Bailey et al., 2006). Another likely explanation for the discrepancy between proviral and plasma viral sequences is that most of the cellular reservoir of HIV-1 is found in anatomically protected and immune-privileged sites and is absent from the peripheral CD4+ T-cells. A combination of assays that can detect and characterize intact proviruses and HIV-1 mRNA in both tissues and blood samples should help identify the likely source(s) of persistent viremia.

### Viral Replication or Proviral Expression as the Major Source of Persistent Viremia?

**FIGURE 1:**
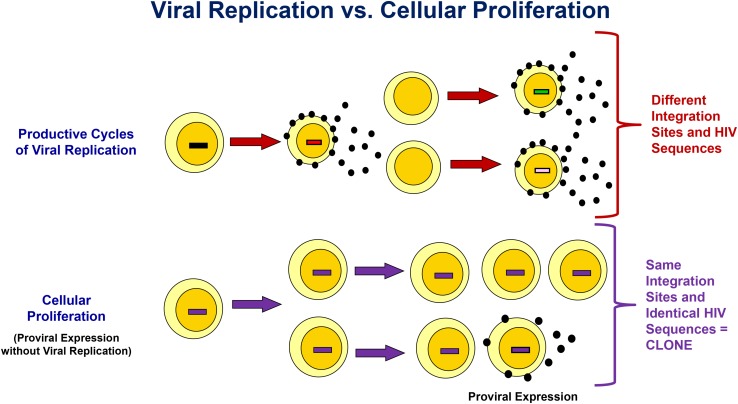
Differentiating Viral Replication From Clonal Expansion with Proviral Expression as the Source of Persistent Viremia on ART. Integration site and HIV sequence analyses can be used to assess the origin of proviral expansion and/or viremia. Productive cycles of viral replication result in both genetic heterogeneity due to errors introduced by reverse transcriptase, and variation in the chromosomal integration site of the HIV provirus. Conversely, clonal expansion results in identical chromosomal HIV integration sites in cell progeny, identical proviral sequences, and identical viral sequences from the subset of cell in the clone that produce virus. See text for more details.

A longstanding debate is whether low-level viremia on ART results from ongoing, complete cycles of viral replication or is from clonally expanded infected T cells that produce virions but that do not infect new cells because they are protected by antiretrovirals (Finzi et al., 1997; Wong et al., 1997; Lorenzo-Redondo et al., 2016). As shown in Figure 1, complete cycles of viral replication give rise to rapid accumulation of mutations attributed to the error-prone nature of reverse transcriptase. An additional consequence of productive cycles of viral replication is multiple integrations of proviral DNA into different locations in chromosomal DNA. Conversely, expansion of infected cells through cellular proliferation produces identical HIV proviral sequences and identical integration sites in cell progeny. Importantly, a subset of these cell progeny can contain transcriptionally active proviruses that produce virions (Figure 1; Bailey et al., 2006; Wagner et al., 2014, Wiegand et al., 2017). For example, Simonetti et al. (2016) reported the detection of a highly expanded CD4+ T-cell clone containing an intact provirus that was a source of persistent viremia on ART.

It has been reported that anatomical sanctuary sites such as the lymph nodes can allow residual viral replication on ART, contributing to maintenance of the HIV reservoir (Lorenzo-Redondo et al., 2016). However, a reanalysis of this data revealed (1) a limited data set after adjusting for PCR resampling and hyper-mutated sequences, (2) limited unique HIV RNA and DNA sequences that were available (median of 5 per sample, range 0–37), (3) sampling time points that may not have taken into account the shifting dynamics of the HIV DNA population within the first year of ART, and (4) inconsistent evidence of viral evolution using more complex analyses (Kearney et al., 2017). These results, along with previous studies reporting no evidence of HIV-1 evolution on suppressive ART in chronically infected adults, as well as in children treated shortly after birth when viral diversity is low, argue against active viral replication being the major source of persistent viremia (Joos et al., 2008; Kearney et al., 2014; van Zyl et al., 2017). Additionally, the presence of invariant sequences and the absence of sequence divergence during prolonged ART, or during and after analytical treatment interruption, is indicative of long-lived cells infected and argues against viral replication. As there have been additional reports of viral replication on ART due to low drug penetration and exclusion of immune cells in anatomical sanctuary sites (Buzón et al., 2010, 2011; Sigal et al., 2011; Hatano et al., 2013; Luo et al., 2013; Patterson et al., 2013; Cardozo et al., 2014; Fletcher et al., 2014; Piovoso and Zurakowski, 2014; Puertas et al., 2014), some residual low-level viral replication on ART cannot be definitively ruled out. Nevertheless, the weight of the evidence discussed above argues against viral replication as the major source of persistent viremia.

Finally, the recent discovery by multiple groups that most of the inducible, infectious virus comes from clonally expanded T-cells argues for cellular proliferation and against ongoing viral replication as the major mechanism for persistence of HIV-1 reservoirs (Lorenzi et al., 2016; Bui et al., 2017; Hosmane et al., 2017). Additional studies are in progress to determine whether most persistent viremia is of clonal cell origin, as has already been described in one instance of an individual with advanced malignancy (Simonetti et al., 2016). Identifying the clonal origin of viremia requires in depth analyses, including full-length single genome sequencing of HIV-1 RNA from plasma and viral outgrowth cultures, and HIV DNA from infected cells, to identify possible clones, with confirmation of clonality by integration site analyses (Palmer et al., 2005; Maldarelli et al., 2014; Bui et al., 2017).

### Clearing Persistent Viremia

Many interventions are being investigated for their ability to clear the HIV-1 reservoir and achieve a functional or sterilizing cure. Among the strategies being studied are: (1) latency reversal to induce viral protein production and expose infected cells to the immune system; (2) engineering immune cells for artificial priming of an HIV-specific immune response or targeted killing of infected cells; (3) gene therapy for alteration of target cell susceptibility to prevent HIV-1 infection; (4) passive immunotherapy with antibodies identified as broadly HIV neutralizing (bnAbs) to clear viremia and infected cells; and combinations of these approaches. These diverse strategies are reviewed elsewhere (Deeks et al., 2016). A promising monoclonal bnAb targeting the CD4 binding site of the HIV-1 envelope (VRC01) has been extensively evaluated for safety, neutralization capacity and pre-existence or development of resistance. Given its high efficacy in neutralizing free virus and the property of bnAbs to promote antibody-dependent cell-mediated cytotoxicity, VRC01 was evaluated in individuals on long-term ART for its effect on persistent plasma viremia and infected cells. Intravenous infusions of VRC01 in individuals on long-term ART did not lead to any change in markers of the reservoir such as cell-associated proviral DNA and RNA, or in levels of persistent plasma viremia (Lynch et al., 2015; Riddler et al., 2018). These disappointing results suggest that persistent virus is either resistant to VRC01 binding or VRC01 effector functions are impaired, such as Fc-mediated clearance or antibody-dependent cellular cytoxicity (ADCC). Work is ongoing to identify the reason(s) for the lack of effect of VRC01 on reservoir markers and on improving the breadth and effector function of bnAbs.

## Unanswered Questions and Concluding Remarks

Whether persistent plasma viremia in individuals on long-term ART consists of infectious virus remains unclear. It has been shown, at least in some instances, that a portion of the persistent viremia produced by infected cell clones is infectious and contributes to rebound virus when ART is stopped (Simonetti et al., 2016; Kearney et al., 2017). Since only an intact *gag* gene is required for virion production and budding (Delchambre et al., 1989), a scenario in which non-infectious virus is released into the plasma and contributes to persistent plasma viremia is also possible. This possibility may help explain the lack of correlation between levels of persistent plasma viremia and the quantity of inducible, infectious virus *ex vivo* (Siliciano et al., 2003; Eriksson et al., 2013; van Zyl et al., 2018). Studies evaluating the ability of virions present in persistent plasma viremia to infect target cells would be useful to address this question.

It is also unclear whether viremia that rebounds following ART interruption is coming from the same source as that producing persistent viremia on ART. Interestingly, a recent study linked clonal proviral populations in infected cells to clonal sequences in rebounding viremia after treatment interruption, showing that infected cell clones are an important viral reservoir (De Scheerder et al., 2019). The report also described preliminary evidence of linkage between virus in plasma and rebound virus in a subset of individuals. Additional studies examining sequences from persistent viremia and rebound virus will further inform the question of the relevance of persistent viremia to rebound off ART.

In conclusion, although unanswered questions remain, remarkable progress has been made toward measuring and characterizing persistent plasma viremia in individuals on ART since it was first reported in 1999. Mounting evidence indicates that persistent HIV-1 viremia on ART largely arises from clonally expanded CD4+ T-cells, although some contribution of ongoing viral replication cannot be excluded. Future and ongoing studies to further characterize clonal populations producing low-level viremia and their mechanisms of escape from immune clearance will be important to achieve a functional cure.

## Author Contributions

All authors listed have made a substantial, direct and intellectual contribution to the work, and approved it for publication.

## Conflict of Interest

JM is a consultant for Gilead Sciences and Xi’an Yufan Biotechnologies, has received research grants to the University of Pittsburgh from Gilead Sciences and Janssen Pharmaceutica, and owns shares in Co-Crystal Pharma, Inc. The remaining authors declare that the research was conducted in the absence of any commercial or financial relationships that could be construed as a potential conflict of interest.
